# Haptic Guidance Needs to Be Intuitive Not Just Informative to Improve Human Motor Accuracy

**DOI:** 10.1371/journal.pone.0150912

**Published:** 2016-03-16

**Authors:** Winfred Mugge, Irene A. Kuling, Eli Brenner, Jeroen B. J. Smeets

**Affiliations:** MOVE Research Institute Amsterdam, Department of Human Movement Sciences, VU University, Amsterdam, Netherlands; University of Chicago, UNITED STATES

## Abstract

Humans make both random and systematic errors when reproducing learned movements. Intuitive haptic guidance that assists one to make the movements reduces such errors. Our study examined whether any additional haptic information about the location of the target reduces errors in a position reproduction task, or whether the haptic guidance needs to be assistive to do so. Holding a haptic device, subjects made reaches to visible targets without time constraints. They did so in a no-guidance condition, and in guidance conditions in which the direction of the force with respect to the target differed, but the force scaled with the distance to the target in the same way. We examined whether guidance forces directed towards the target would reduce subjects’ errors in reproducing a prior position to the same extent as do forces rotated by 90 degrees or 180 degrees, as it might because the forces provide the same information in all three cases. Without vision of the arm, both the accuracy and precision were significantly better with guidance directed towards the target than in all other conditions. The errors with rotated guidance did not differ from those without guidance. Not surprisingly, the movements tended to be faster when guidance forces directed the reaches to the target. This study shows that haptic guidance significantly improved motor performance when using it was intuitive, while non-intuitively presented information did not lead to any improvements and seemed to be ignored even in our simple paradigm with static targets and no time constraints.

## Introduction

We rely on vision and proprioception to provide information about the position and orientation of our body. Our central nervous system integrates this into a single percept [1,2]. When executing movements humans make both random and systematic errors. Some of the systematic errors probably arise from biases between proprioceptive and visual estimates [3–6]. Can we reduce these errors by providing forces that scale with the distance from the goal?

Research into reaching movements in force fields has primarily focused on measuring movement errors [6,7] and the way we cope with them through error correction [8,9], motor adaptation [10] or motor learning [11]. Perturbation forces introduce errors that are corrected through feedback mechanisms and (in subsequent repetitions) through feedforward mechanisms. It has been shown that the position sense is not disturbed by external forces at the end-effector [6,12], which implies that forces can be applied in haptic robot interaction without distorting the position percept of the human operator. The relation between forces and position errors is very relevant for telemanipulation, a technique used for situations in which a human operator is required (in order to judge and resolve unexpected situations) while physical presence is undesirable (e.g. due to hazardous environments) [13,14]. In telemanipulation, an operator controls a remote slave device through physically interacting with a master that serves as an input device. Providing the operator with force feedback from the interaction between the slave and its environment yields a reduction in task completion time [15–17], control effort [17], and cognitive workload [18].

Even with perfect transparency, where the forces on the operator are an exact copy of the interactive forces between the slave robot and its environment, performance will be limited by errors of the human operator. A promising recent development that may circumvent this limitation is haptic shared control, where the operator and an automatic controller act and exchange information in a simultaneous and continuous way [19,20]. Haptic shared control is an approach in which an assisting system continuously communicates a calculated ideal control input to the human operator through forces, which can be seen as a way of combining automation and manual control. It has proven itself in car driving, reducing lane keeping variability by 30% while reducing visual demand [21]. Although lane keeping can be fully automated, it is preferred to keep the driver in charge and engaged due to unpredictability and legal responsibility issues [20,22–24]. In haptic shared control the torque from the controller informs the driver how to correct the current steering angle to the optimal one without ever overpowering the driver. That is an essential property of haptic shared control: the driver is always able to override the additional torque [20].

Experiments in other areas have also revealed benefits of haptic shared control. Rosenberg [25] presented additional passive guiding forces—called virtual fixtures—that assisted the operator during a teleoperated peg-in-hole task, improving task performance. Boessenkool et al. [26] showed that haptic shared control during free air movements in a bolt-spanner task reduced errors, control effort and cognitive workload. Dvorkin et al. [27] applied various haptic manipulations to reaching movements of patients with traumatic brain injury, and found superior performance in a haptic nudge condition in which a force of 1N was briefly applied in the direction of the target when the subject stopped moving. The above-mentioned improvements may be based on the additional information that the haptic shared control communicates to the human operator, but there is an alternative explanation.

Forces that guide the operator to the target, normally do not only provide information about the direction and distance to the target (through the direction and magnitude of the force), but also feel intuitive: the operator can directly follow the forces to reach the target. Our study examined whether haptic guidance needs to be intuitive to be useful, or whether it is the additional information about the target’s position with respect to the hand that improves the operator’s performance in a position reproduction task.

Three horizontal force fields were centered on the target; the relation between the direction of the force and the direction to the target differed between the fields, but the force scaled with distance in the same way. The three force fields thus provide exactly the same information to the subjects. Although the movements may be less straight in less intuitive force fields and subjects may require more time to extract the information than from more intuitive ones, if it is the additional information that the haptic shared control provides that is responsible for the reduction of motor errors, then all force fields will reduce end point errors to the same extent. If it is not only the information that matters, but also how intuitively it is presented (the ease with which it can be used), then only assisting forces will reduce motor errors. We therefore examined whether in the absence of time constraints, subjects would reproduce a prior position equally well with 90 degrees or 180 degrees rotated guidance as with guidance directed towards the target.

## Methods

### Subjects

This study consisted of 2 experiments; ten subjects (all right-handed, 30.8 SD 9.3 years, 6 male and 4 female) took part in Experiment 1 and ten subjects (all right-handed, 31.5 SD 9.1 years, 4 male and 6 female) in Experiment 2. As 4 subjects performed both the experiments (of which 2 are co-authors of this paper), 16 subjects participated in total. The study is part of an ongoing research program that has been approved by the ethics committee of the Faculty of Human Movement Sciences of VU University. All subjects were naïve about the purpose of the experiments (the two co-authors performed the experiments before being informed about the details) and gave their written informed consent prior to participation.

### Experimental setup

**Fig 1:**
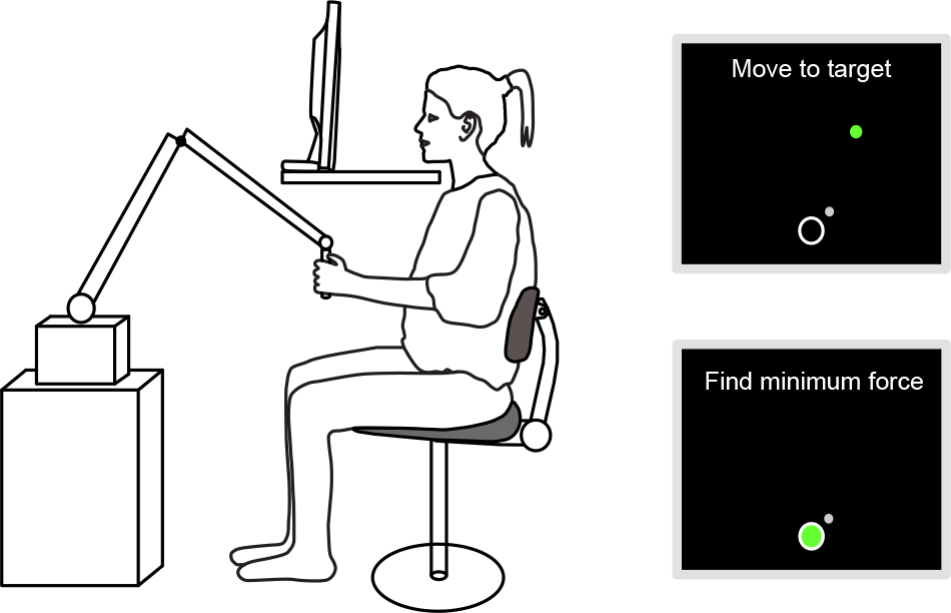
Experimental setup. Subjects made reaches to a visual/haptic goal while holding a haptic device (PHANToM Premium 3.0/6DoF). Right panel shows the onscreen visualizations with a target indicator in Experiment 1 (top) and without a target indicator in Experiment 2 (bottom).

Subjects made self-paced reaches from a starting position, centered close to the chest, to two targets in the horizontal plane (located 0.25m away from the starting position and 0.10m left or right of the sagittal plane) while holding the end effector of a PHANToM Premium 3.0/6DoF (Geomagic). Subjects fully enclosed the end effector of the haptic device in their hand with their thumb operating the button to indicate the completion of the reach (Fig 1). Vision of the hand and robot was obstructed by a table and a monitor (33.8x27.0cm, 1280x1024 pixels, 60 Hz) in front of the subject. Specialized software was used for the onscreen visualizations and data acquisition at 300 Hz (D-Flow, MotekForce Link). The monitor always showed a 15 mm radius circle (line thickness 4 mm) as starting point, and sometimes showed a 10 mm radius disk as target (Experiment 1 only) and a 9 mm radius disk as cursor (indicating the hand position). The movement of the cursor corresponded to the horizontal component of the hand movement. In trials in which no cursor was presented, the cursor reappeared when the hand approached the starting position after the reach, to help the subject accurately locate the starting position. When at the starting position, pushing the button initiated the next trial with the next force field and visual indicator settings.

To prevent the subject’s hand from drifting vertically from the target plane, either the color of the target (Experiment 1) or that of the starting point (Experiment 2) indicated whether the height was correct: green if the height was within 30 mm of the target plane, red if above and blue if below.

### Guidance conditions

**Fig 2:**
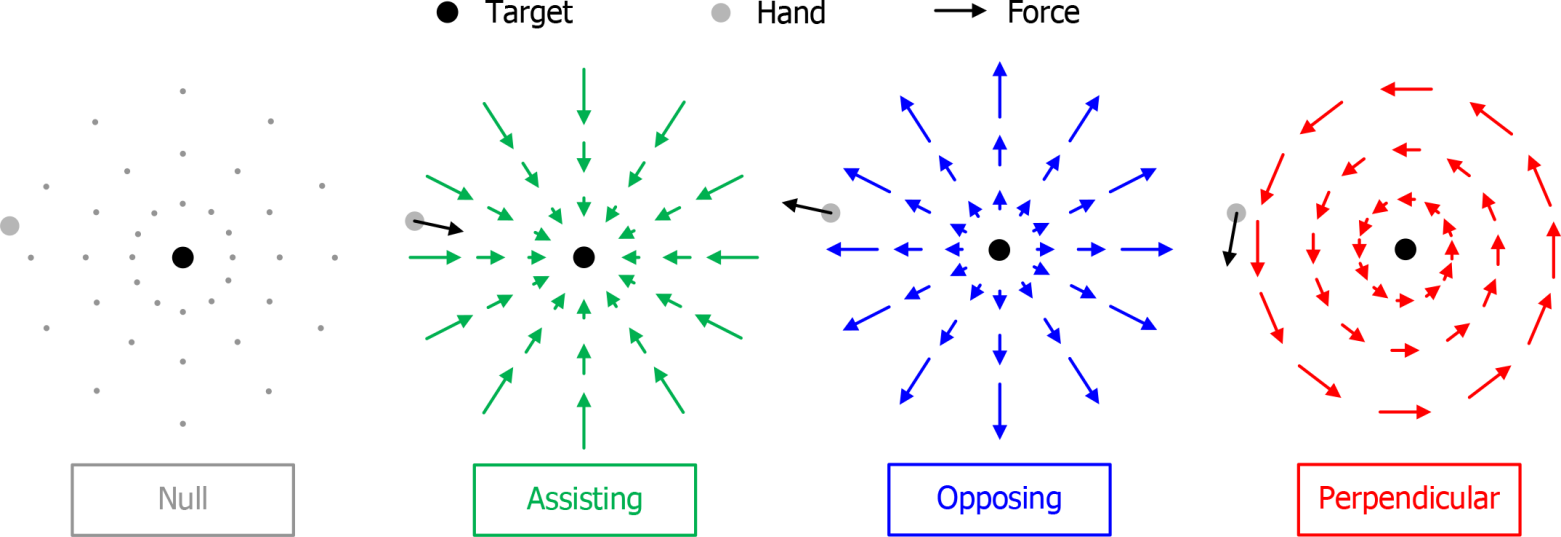
Guidance conditions. The Null condition without forces was only used in Experiment 1; the three conditions with force fields were used in both experiments. The Assisting (most intuitive), Opposing and Perpendicular (least intuitive) guidance proportionally scale the force with the distance to the target, effectively providing the same information regarding the location of the target.

All force fields (50 N/m) were centered on the target and capped at 5 N for distances of more than 10 cm from the target to prevent excessive displacements from the starting position when initiating the force field. The forces were independent of the vertical position of the handle and no forces were exerted in vertical direction. All force fields (all conditions except for the Null condition) provided the same information to the subject; the magnitude of the force indicated the distance from the target and the direction of the force indicated the direction to the target. The only difference is that in two force fields the direction of the force was rotated with respect to the direction to the target (Fig 2):

1. Null condition (no guidance, only Experiment 1)
2. Assisting guidance condition (guidance directed towards the target, 0 degrees rotated)
3. Opposing guidance condition (guidance directed away from the target, 180 degrees rotated)
4. Perpendicular guidance condition (guidance directed perpendicular to the target direction, 90 degrees rotated)

Assisting guidance is an intuitive implementation of guidance, which is known to improve performance [21,25–27]. To attain the target, one can simply move along with the force. Opposing guidance is less intuitive as one will have to oppose the force to move in the direction of the target. Perpendicular guidance presents a complex relationship between the force and the required movement.

### Task instruction and experimental protocol

In Experiment 1, subjects were instructed to move to a visual target. In Experiment 2, subjects were instructed to accurately locate the center of the force field, at which the force was zero. In both experiments, subjects were instructed to move to the target as accurately as possible, with no time constraints, and push the button when the target was attained. They were informed that the target location did not include a vertical component, but were nevertheless requested to try to maintain a green height indicator throughout the movement.

In Experiment 1, the reaches were performed in pairs towards the same visual target, first with a visible cursor (visual trial) and then without (blind trial). In Experiment 2, only haptic trials were performed. They were similar to the blind trials of Experiment 1, but the target was the center of the force field rather than a visible target and they were not alternated with visual trials with a visible cursor. Therefore, subjects could not use memory of (a movement towards) a visual target, but were forced to use the haptic information. Note that there was no Null condition in Experiment 2, as this condition would not provide any information on target location. In both experiments there were separate blocks of trials for all conditions. These blocks were presented to the subject in random order. Before presenting each block of trials with a certain force field, the force field was explained to the subject and he/she performed several training trials. Experiment 1 consisted of 192 trials (2 visual/blind conditions x 4 force fields x 2 target locations x 12 repetitions) and Experiment 2 of 72 trials (3 force fields x 2 target locations x 12 repetitions).

### Analyses

The 2D positions at which the subjects pushed the button were measured and three performance measures were taken:

- Accuracy (systematic error) was calculated as the distance between the mean end point and the target.
- Precision (random error) was calculated as the average distance between the individual end points and the mean end point.
- Reaching time was calculated as the time between the button push at the starting position and the button push at the end point. Note that it was explicitly stated to the subjects that time was not a factor.

Additionally, the path length was analyzed to judge whether the trajectories were more straight (shorter path length) in certain conditions. The accuracy, precision, reaching time, and path length in the visual and blind trials (Experiment 1), and in the haptic trials (Experiment 2), were all assessed separately. Repeated measures ANOVA’s were performed on accuracy, precision, reaching time, and path length with a significance level at p<0.05. Subsequent post hoc analyses were Bonferroni corrected and in case sphericity was violated according to Mauchly’s test for sphericity the lower bound estimate was used.

## Results

### Experiment 1

**Fig 3:**
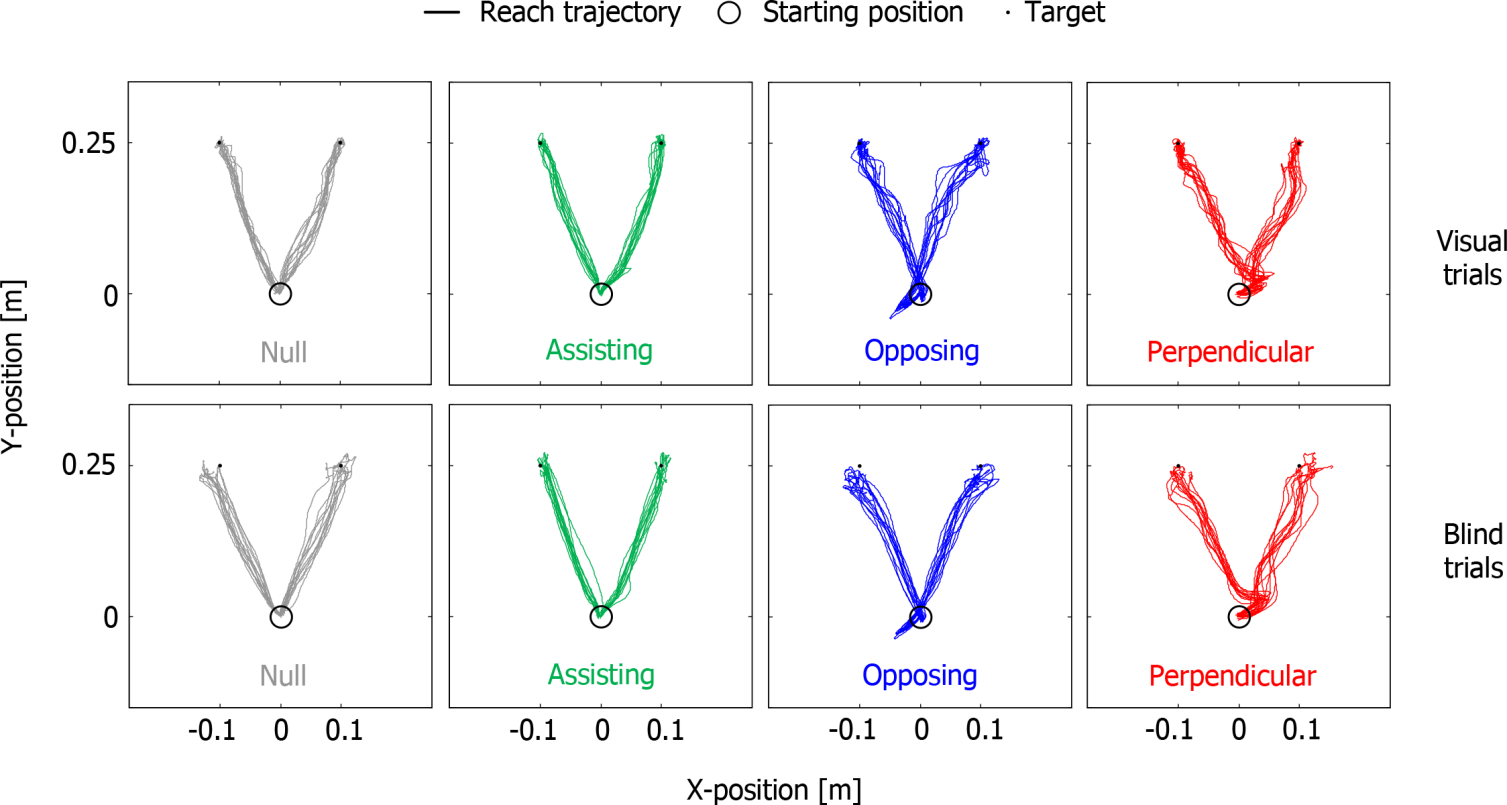
All reach trajectories of a typical subject in Experiment 1. Reaches were quite straight, with only evident effects of the force fields at the start of the movement when the forces were the strongest and the subjects experienced a transient onset of the force field. Top panels show visual trials; bottom panels show blind trials.

**Fig 4:**
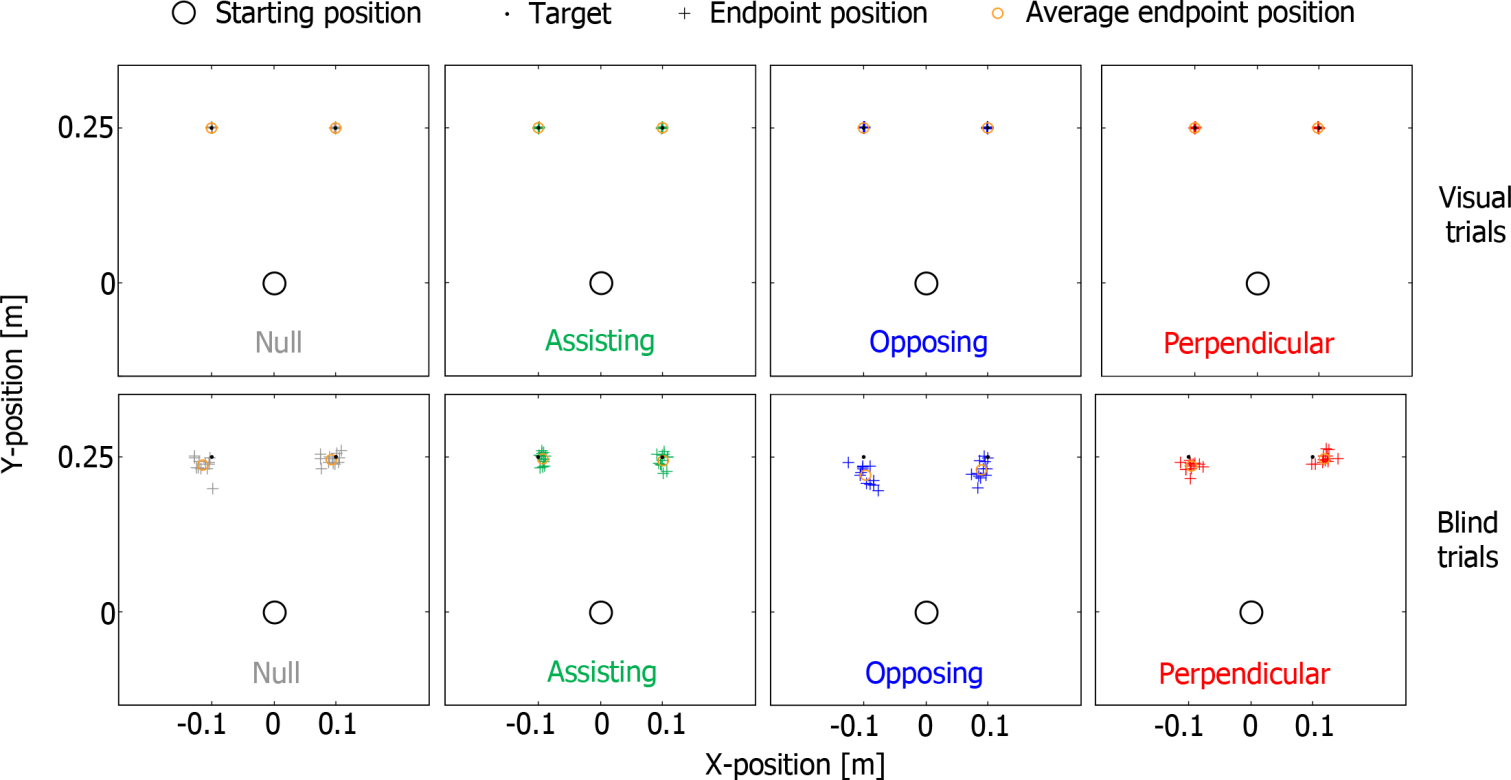
End points of all the reaches of Fig 3. The black dots represent the targets, the plusses the end points of individual trials and the orange circle the average end point position for each target. Top panels show visual trials; bottom panels show blind trials.

Subjects made consistent almost straight goal-directed reaches to the targets. For the three conditions with force fields, the early parts of the trajectories (close to the starting position, see Fig 3) reflect initial displacements due to the onset of the force field at the starting position. In the visual trials the subjects accurately attained the targets for all four conditions. In the blind trials the end points of the movements (Fig 4) show larger random errors and systematic errors typically fell short of the targets. The smaller precision in the absence of vision indicates that the forces themselves were not the decisive factor in attaining the end points.

**Fig 5:**
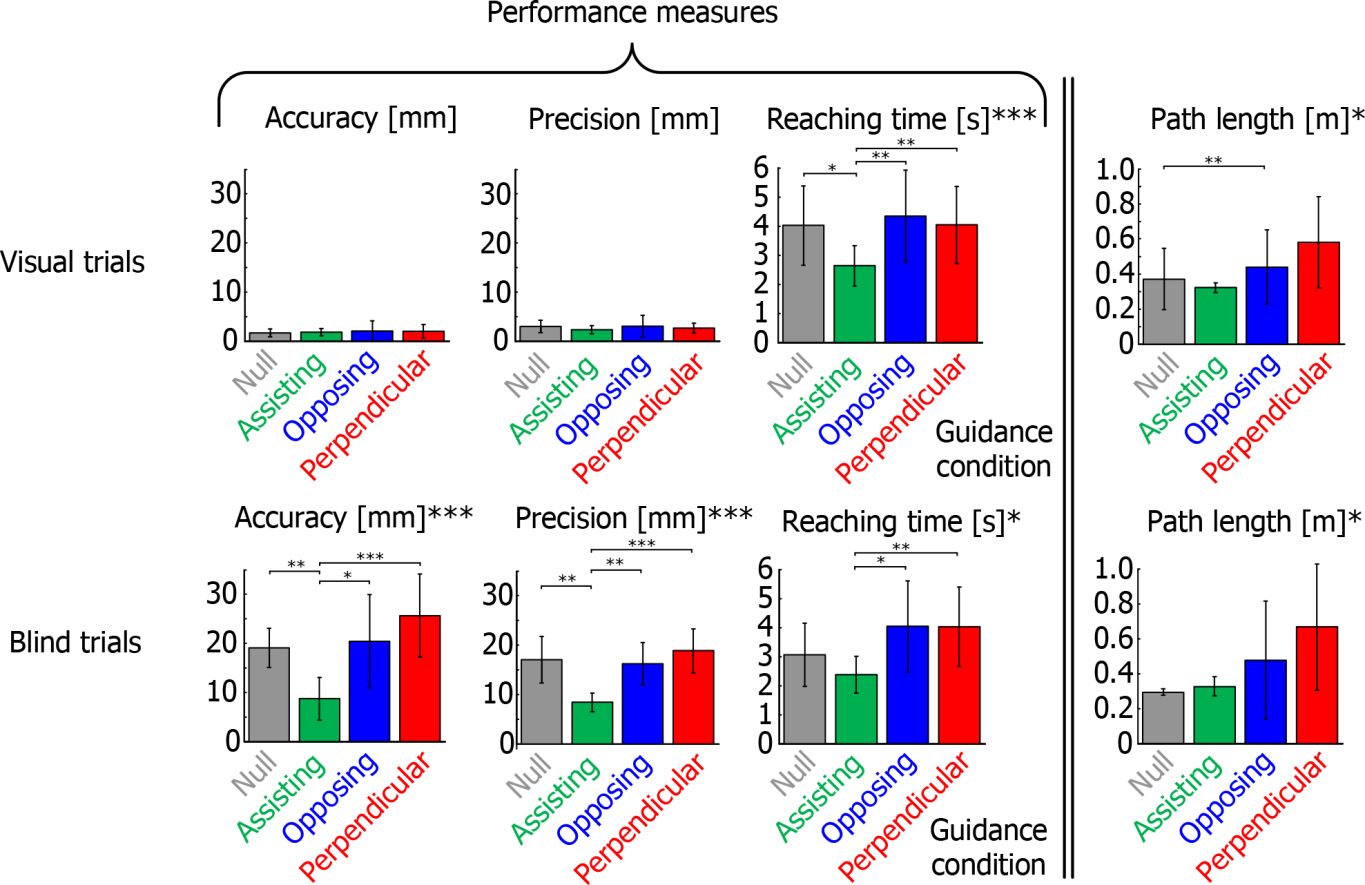
Mean performance measures accuracy, precision and reaching time, as well as the mean path length of the reaches in Experiment 1. Error bars indicate standard deviation over subjects and asterisks indicate significance (*: p<0.05, **: p<0.01, and ***: p<0.001). The assisting guidance condition improved the subjects’ performance with respect to all other guidance conditions: reaches were completed faster (both types of trials) and with increased accuracy and precision (blind trials).

In visual trials, we found a significant main effect of guidance condition on reaching time (Fig 5; F_3,27_ = 14.149, p<0.001) and path length (F_1,9_ = 7.085, p = 0.026), without any effect on accuracy and precision (F_1,9_ = 0.231, p = 0.642; F_1,9_ = 1.113, p = 0.319 respectively). Post-hoc tests on the reaching times revealed that the visual trials with Assisting guidance were completed significantly faster than those in any other guidance condition, while errors were not larger. Post-hoc tests on the path length only revealed significantly longer path lengths in the Opposing condition in comparison with the Null condition.

In blind trials, we found significant main effects of guidance condition on accuracy, precision, reaching time and path length (F_3,27_ = 12.323, p<0.001; F_3,27_ = 12.498, p<0.001; F_1,9_ = 9.352, p = 0.014; F_1,9_ = 7.034, p = 0.026, respectively). Post-hoc tests revealed that the blind trials with Assisting guidance were significantly more accurate and precise than the blind trials of any other guidance condition. In addition, movements with Assisting guidance in blind trials were completed significantly faster than those with Opposing and Perpendicular guidance, but were not significantly different from those of the Null condition (p = 0.515). Post-hoc tests on the path length did not reveal any significant differences.

Experiment 1 shows that only assisting guidance reduced subjects’ errors with respect to no guidance at all (Null condition). For the Perpendicular as well as the Opposing guidance, the errors were larger than with intuitive guidance, showing that it is not only the information that leads to the reduction of errors. Surprisingly, the information did not appear to contribute at all, as the errors in the two non-intuitive conditions tends to be larger than without guidance.

### Experiment 2

**Fig 6:**
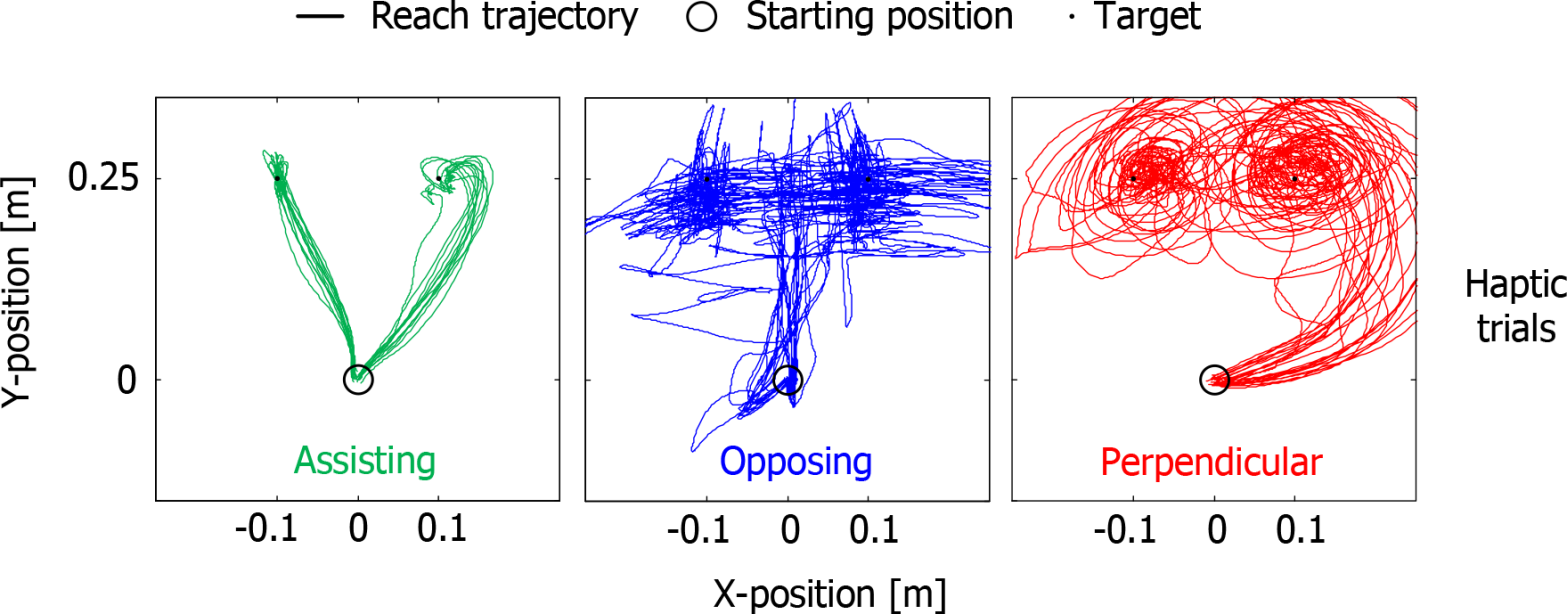
All reach trajectories in Experiment 2 from the same subject as in Fig 3. In Experiment 2, without a preceding visual trial and without visual targets, subjects made more explorative movements than in Experiment 1.

Subjects’ movements differed considerably from those in the blind trials in Experiment 1. Subjects now made explorative movements to determine the center of the force fields (Fig 6).

**Fig 7:**
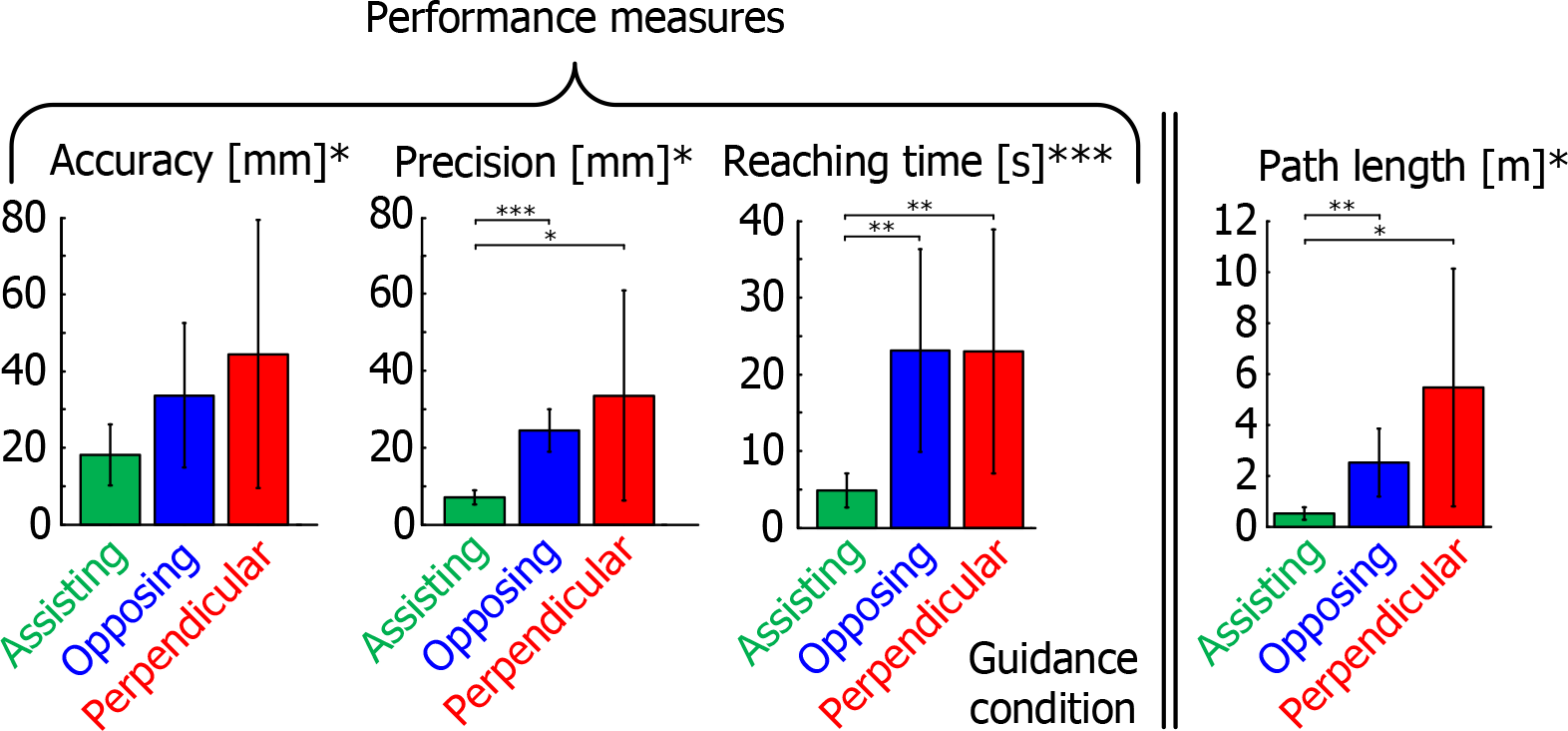
Mean performance measures accuracy, precision and reaching time, as well as the mean path length of the reaches in Experiment 2. Error bars indicate standard deviation over subjects.

We found significant main effects of guidance condition on accuracy, precision, reaching time, and path length (Fig 7; F_2,18_ = 5.204, p = 0.016; F_1,9_ = 7.400, p = 0.024; F_2,18_ = 12.427, p<0.001; F_1,9_ = 8.102, p = 0.019, respectively). Post-hoc tests revealed that with Assisting guidance movements were completed significantly faster, with shorter path lengths, and more precisely than with Opposing and Perpendicular guidance, but there were no significant differences in accuracy between the conditions. Thus, in the haptic trials the same main effects were present as in the blind trials of Experiment 1.

## Discussion

In Experiment 1, haptic guidance only improved performance when the force field provided guidance forces towards the target (Assisting), despite the fact that two other force fields (Opposing and Perpendicular) provided the same amount of information regarding the position of the target. In Experiment 2, the benefit of intuitive guidance on the performance measures, accuracy, precision and reaching time, persisted. In the less intuitive conditions the movements had longer path lengths. Possibly, in those conditions, the forces disturbed the subject’s reach. Alternatively the subjects might have needed more time and force inputs to extract the information from the Opposing and Perpendicular forces, because the movements appeared to be more exploratory. Apparently, it is essential to provide the guidance in a way that directly helps perform the task, perhaps because people intuitively expect a force to be helpful in these circumstances, but it could also be that because such guidance assists performance it seems to them to be trustworthy, thereby making its use intuitive.

One might argue that subjects just need more practice to learn to use the less intuitive guidance implementations. We therefore explored whether we could find any learning effects in the accuracy and precision in the blind trials of Experiment 1 and the haptic trials of Experiment 2, but they were not found (not shown). For Experiment 1, this suggests that the subjects chose to ignore the information that was present in the guidance and trust their memory of the position, based on the preceding visual trial. Subjects indicated in an informal debrief of the experiment that they were unaware that only two target positions were used, some indicated to have perceived many more. The better performance in the blind trials (with preceding visual trials) than in the haptic trials reflects the benefit of the memory of the position from the preceding trial. The path lengths were also considerably longer without prior knowledge of the target location (compare haptic trials in Experiment 2 with blind trials in Experiment 1), indicating more exploratory movements in the haptic trials (see Fig 6). The main finding is that guidance did not reduce the errors unless it assisted the movements, despite the lack of target dynamics and time constraints, and despite subjects taking more time in the other conditions.

Another possible explanation for our data is that the positive stiffness that the assisting guidance provides reduces variability due to motor noise (“Guidance directly influences the execution errors, either reducing or enlarging these errors” [28]). However, the same reasoning would predict that performance would be worse with the negative stiffness of the Opposing condition, as compared to the Null and the Perpendicular condition, which it is not, indicating that the results cannot easily be explained through the combined mechanical properties of the human and the robot.

The much smaller errors in the visual trials than in the blind trials, even with Assisting guidance, shows that the final position is primarily (maybe even exclusively) determined by (inaccuracies of) the subject. If the forces near the target would drag the subject to the target then the accuracy would not improve (much) with visual feedback. Moreover, if the forces near the target would move the subject to it when assisting, then subjects would have been able to have the forces move them away from it when opposing. The extent to which one can know that one is at the target does not depend on the kind of force field, because whenever there was a noticeable force or resulting movement the subject could know that they were not on target. Apparently, there was a considerable range within which the applied forces were too low to overcome the subject’s impedance and move the hand. If these subtle forces are difficult to perceive, what makes Assisting guidance so special? Why would finding the bottom of a valley be easier than finding the top of a hill?

Subjects might trust the guidance more when it is assisting them, because they feel that they are in agreement with the system, collectively moving towards the target. This feels intuitive in the sense that you instantly understand the system’s intentions. At the end point the subjects may therefore be more inclined to ‘listen’ to what the guidance is telling them. On top of that, the implementation of the Assisting guidance allows subjects to give the authority to the guidance by lowering their stiffness. This ties closely into optimal control [29] where the subject may be aware that the noise of the robot is below that of his/her motor noise. The destabilizing property of the Opposing condition may evoke behavior that preserves stability, such as cocontraction that may mask the effects of the guidance through signal-dependent noise [30]. Yet, stability alone cannot fully explain the results as the Perpendicular condition has no radial (in- or outward) stiffness and did not improve performance with respect to the Null or Opposing condition either. Subjects may have been too stubborn to reduce their stiffness in the Opposing and Perpendicular conditions. Possibly the fact that giving way to the guidance by lowering one’s stiffness in itself does not work towards achieving the target, makes the operator unwilling to lower his or her stiffness in order to better extract the information.

Our finding that informative but non-intuitive guidance did not improve performance may be useful in applications such as rehabilitation. So far, methods that use interactive visuo-haptic environments have potential advantages over more standard forms of clinical care [27]. Therapy robots typically move a limb through a pre-specified trajectory where deviations from this trajectory result in forces toward this trajectory [28,31]. These forces range from soft guidance with assisting forces to hard guidance with tunnel walls around the pre-specified trajectories. In the latter, the subject can be fully passive and have the robot perform the movement [32,33]. Hitherto, motor learning with haptic guidance has not yet revolutionized the field. In fact, there are examples where learning of novel visuomotor transformations with haptic guidance added to visual feedback was poorer than without haptic guidance [28, 34]. The motor system adapts to the environment including the haptic guidance, creating aftereffects that interfere with performance when the haptic guidance is taken away [35–37]. Our finding may indicate that interaction with haptic guidance occurs on a subconscious level, which may be a second cause for the limited benefit of haptic guidance in motor learning.

## Conclusion and Implications

Subjects only improved performance on a position reproduction task with assistive haptic guidance when the additional haptic information was presented in an intuitive manner. The same information presented non-intuitively was ignored, even in our paradigm with static targets and no time constraints. Moreover, the effects of accuracy, precision, reaching time, and path length persisted when performing a purely haptic task, indicating that the effects were not specific to movements towards remembered positions from visual trials.

Intuitive implementation of guidance is important and likely ties into effectively combining the capabilities and limitations of the human and the machine (optimal control).

## Supporting Information

S1 Data Raw_Data_Exp1.zip Supplementary Information—Raw Data Experiment 1 (ZIP)

S2 Data Raw_Data_Exp2.zip Supplementary Information—Raw Data Experiment 2 (ZIP)
